# Brown Tumor as a Result of Hyperparathyroidism in an End-Stage Renal Disease Patient

**DOI:** 10.1155/2011/415476

**Published:** 2011-10-26

**Authors:** Jesse M. Jakubowski, Ines Velez, Shawn A. McClure

**Affiliations:** Department of Oral and Maxillofacial Surgery, Nova Southeastern University, FL 33314-7796, USA

## Abstract

A 49-year-old male with known history of end-stage renal disease (ESRD) presents with an intraoral exophytic mass of the right mandible. This lesion was given a histologic diagnosis of a Brown tumor. *Purpose*. To allow physicians to include this lesion in a differential diagnosis when evaluating patients with primary, secondary, or tertiary hyperparathyroidism.

## 1. Introduction

Hyperparathyroidism (HPT), the overproduction of parathyroid hormones, results in an increased release of calcium from bone which directly affects the skeletal system. Calcium's concentration in plasma is increased and its absorption by the kidneys and intestine is altered. This leads to skeletal demineralization and allows for bone to be replaced by multinucleated giant cells believed to be osteoclasts weakening the skeletal structure [1]. 

Primary HPT is caused by hypersecretion of the parathyroid hormone (PTH), usually due to hyperplastic or tumoral changes of one of the four parathyroid glands. The result is hypercalcemia. Secondary HPT is usually due to end-stage renal disease (ESRD). There is renal loss of calcium, and with normal parathyroid glands, elevation of PTH occurs in response to the hypocalcemia. Tertiary HPT appears in patients with longstanding secondary disease, as hyperplasia of parathyroid glands and loss of response to the level of calcium in serum [2].

The hallmark of PTH excess is increased osteoclastic activity with bone resorption. Cortical and trabecular bone are lost and replaced by loose connective tissue. In some instances, collections of osteoclasts, reactive giant cells, and hemorrhagic debris form a distinct mass, termed a brown tumor of hyperparathyroidism [3]. 

## 2. Case Report

A 49-year-old African American male presents with a chief complaint of right mandibular pain, edema, and intraoral bleeding at night. This condition has persisted for 12 months with a gradual increase in size and pain. This patient has medical history significant for ESRD, for which he undergoes dialysis three times per week for the past 10 years, congestive heart failure, anemia, diabetes mellitus, and atrial fibrillation. He has been prescribed oral calcium and vitamin D supplements which he has not been compliant with.

**Figure 1:**
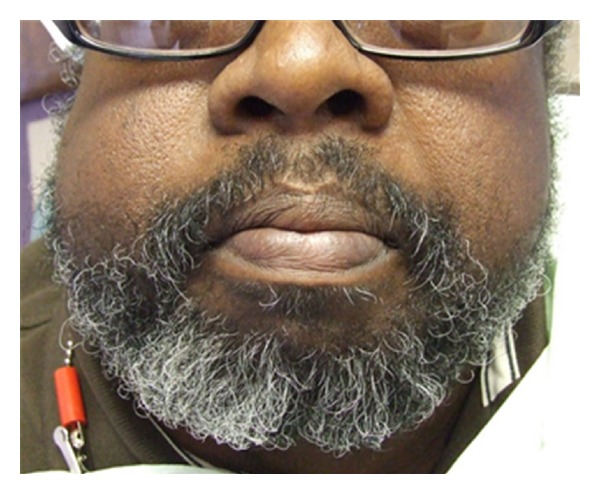
Extraoral presentation.

**Figure 2:**
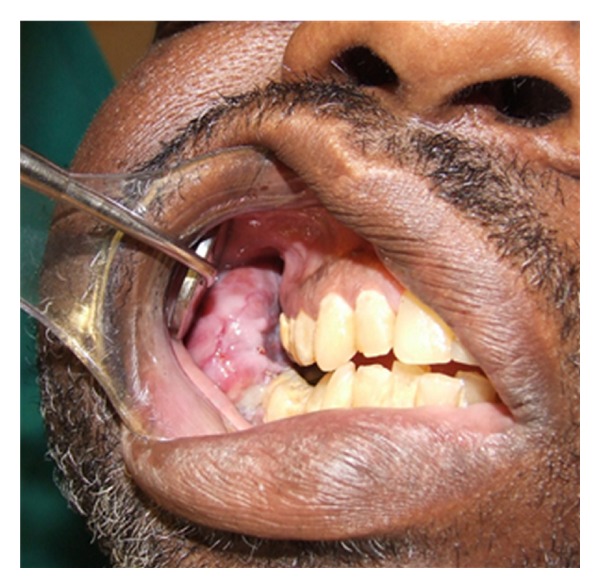
Intraoral expansion of the mandible.

Extraoral examination reveals facial asymmetry with right facial enlargement (Figure 1) which is soft to palpation and mildly tender, no trigeminal nerve paresthesia was noted. Intraoral examination shows a large soft tissue mass measuring 8 cm × 2 cm × 4 cm extending from right mandibular second premolar to 2 cm past right mandibular third molar (Figure 2). There was no buccal or lingual expansion of bone noted clinically. The first molar was mobile and the second and third molars were free floating lingually in the soft tissue mass. Bleeding and pain were noted upon palpation of mass and associated teeth. 

**Figure 3:**
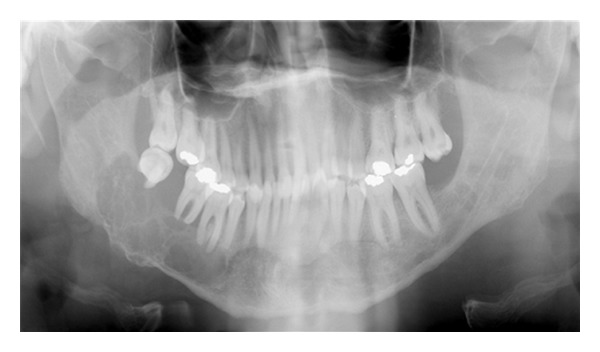
Panoramic radiography showing a multilocular radiolucency of the right mandible.

 Panoramic radiograph shows a well-defined multilocular radiolucent lesion in the right mandibular body. This lesion extends from one centimeter posterior to the third molar to the second premolar in a horizontal dimension and from the inferior border of the mandible to the alveolar ridge in a vertical dimension. The mandibular second and third molars are displaced and the inferior alveolar nerve canal is obliterated (Figure 3). 

With clinical and radiographic diagnosis of brown tumor versus odontogenic tumor, an incisional biopsy was performed and a specimen from the central area of the soft tissue mass measuring 1.2 cm × 0.6 cm × 0.5 cm and sent to pathology in formalin. H&E stain showed numerous multinucleated giant cells in a hypercellular fibrous background with hemosiderin pigmentation. A histological diagnosis of brown tumor of hyperparathyroidism was rendered.

## 3. Discussion

ESRD refers to bilateral, progressive, chronic deterioration of nephrons, the functional unit of the kidney. The disease results in uremia and can lead to death [4]. Decreased glomerular filtration occurs with reduced nephron function, which results in decreased 1,25-dihydroxyvitamin D synthesis by the kidney leading to decreased calcium absorption by the gut. Consequently an increased level of serum phosphate is also seen. Phosphate is the driving force of bone mineralization, excess phosphate tends to cause serum calcium to be deposited in bone, leading to a decreased serum calcium level and structurally deficient bones. In response to low serum calcium, the parathyroid glands are stimulated to secrete PTH, which results in secondary HPT [4]. The patient becomes hypocalcemic and hyperphosphatemic, opposite of primary HPT [5]. 

A variety of bone disorders are seen in ESRD; these are collectively referred to as renal osteodystrophy [4]. In some cases like the present one, HPT is diagnosed by the presence of osteolytic lesions called brown tumors. Only about one in five patients with HPT has radiographically observable bone changes. The major manifestations of HPT are as follows.

1. Subtle erosions of bone from the subperiosteal surfaces of the phalanges of the hands.
2. Demineralization of the skeleton resulting in an unusual radiolucent appearance.
3. Osteitis fibrosa cystica in localized regions of bone loss produced by osteoclastic activity, resulting in a loss of all apparent bone structure.
4. Brown tumors which occur late in the disease and in about 10% of cases.
5. Pathologic calcifications in soft tissues that have a punctate or nodular appearance and occur in the kidneys and joints.
6. In prominent HPT the entire calvarium has a granular appearance caused by the loss of central trabeculae and thinning of the cortical tables [6].

Brown tumors are caused by alterations in the trabecular bone pattern, demineralization, and replacement by loose connective tissue. They present as uni/multilocular radiolucencies, and/or mixed lesions with bone expansion, bone deformity, tooth mobility, and loss of lamina dura [7]. The radiographic manifestations are in many cases incidental findings and may be multiple. Demineralization and thinning of cortical boundaries often occur in the jaws in cortical boundaries such as the inferior border, mandibular canal, and the cortical outlines of the maxillary sinuses. The density of the jaws is decreased, resulting in a radiolucent appearance that contrasts with the density of the teeth. The teeth stand out in contrast to the radiolucent jaws. A change in the normal trabecular pattern may occur, resulting in a ground-glass appearance of numerous, small, randomly oriented trabeculae [6].

Histologically Brown tumor of HPT is similar to giant cell tumor: multinucleated giant cells in a background of spindle cell proliferation containing a large amount of hemosiderin [3, 8, 9]. 

The best diagnostic method for secondary HPT is parathyroid immunoassay and confirmation of ESRD. Further workup with osseous scintigraphy (using technetium 99 m—methylene diphosphonate) may be indicated to locate other areas of osseous metabolic disease as discussed by Prado et al. [10].

The treatment of a Brown tumor is mainly pharmalogic by treating the underlying HPT; however, surgical excision is sometimes necessary. Triantafillidou et al. advocate curettage of the lesion and wound packing allowing for secondary healing in addition to adjunctive treatment of underlying disease [11]. HPT is commonly treated with calcium, vitamin D, and controlled dialysis, although renal transplant or subtotal parathyroidectomy may also be options. Since bone healing in these patients is compromised, HPT must be controlled prior to successful surgical bone reconstruction [5, 12].

## 4. Conclusion

ESRD is a worldwide problem that continues to increase; these patients may have many serious medical problems, and physicians must know how to manage them [13]. ESRD can lead to HPT, which has several manifestations that can be seen in clinical and radiographic exams. The physician should be aware of the clinical manifestations and radiographic appearances of these lesions to allow for early treatment of the underlying diseases and improvement of overall prognosis.
